# Individualized treatment response prediction of dialectical behavior therapy for borderline personality disorder using multimodal magnetic resonance imaging

**DOI:** 10.1002/brb3.1384

**Published:** 2019-08-14

**Authors:** Mike M. Schmitgen, Inga Niedtfeld, Ruth Schmitt, Falk Mancke, Dorina Winter, Christian Schmahl, Sabine C. Herpertz

**Affiliations:** ^1^ Department of General Psychiatry Medical Faculty Heidelberg Heidelberg University Heidelberg Germany; ^2^ Department of Psychosomatic Medicine and Psychotherapy Central Institute of Mental Health Medical Faculty Mannheim Heidelberg University Mannheim Germany; ^3^ Center for Mental Health Odenwald District Healthcare Center Erbach Germany

**Keywords:** functional MRI, machine learning, multimodal data analysis, prediction of treatment response, random forest, structural MRI

## Abstract

**Introduction:**

Individualized treatment prediction is crucial for the development and selection of personalized psychiatric interventions. Here, we use random forest classification via pretreatment clinical and demographical (CD), functional, and structural magnetic resonance imaging (MRI) data from patients with borderline personality disorder (BPD) to predict individual treatment response.

**Methods:**

Before dialectical behavior therapy (DBT), 31 female patients underwent functional (three different emotion regulation tasks) and structural MRI. DBT response was predicted using CD and MRI data in previously identified anatomical regions, which have been reported to be multimodally affected in BPD.

**Results:**

Amygdala and parahippocampus activation during a cognitive reappraisal task (in contrasts displaying neural activation for emotional challenge and for regulation), along with severity measures of BPD psychopathology and gray matter volume of the amygdala, provided best predictive power with neuronal hyperractivities in nonresponders. All models, except one model using CD data solely, achieved significantly better accuracy (>70.25%) than a simple all‐respond model, with sensitivity and specificity of >0.7 and >0.7, as well as positive and negative likelihood ratios of >2.74 and <0.36 each. Surprisingly, a model combining all data modalities only reached rank five of seven. Among the functional tasks, only the activation elicited by a cognitive reappraisal paradigm yielded sufficient predictive power to enter the final models.

**Conclusion:**

This proof of principle study shows that it is possible to achieve good predictions of psychotherapy outcome to find the most valid predictors among numerous variables via using a random forest classification approach.

## 1 INTRODUCTION

Although dialectical behavior therapy (DBT) is the currently best‐established psychosocial treatment for borderline personality disorder (BPD; Cristea et al., 2017; Kliem, Kroger, & Kosfelder, 2010; Stoffers et al., 2012), it is unclear who will respond best to this therapy. Since psychotherapy is quite expensive, a prognostic tool to predict which patient will benefit from DBT is desirable. However, so far only few investigations have been made to gain a better understanding of individual predictors of therapy response in BPD.

In anxiety disorders, it has been shown that functional and structural magnetic resonance imaging (MRI) data in addition to variables such as clinical characteristics and demographics (CD) or electroencephalography (EEG) can provide useful information for models predicting treatment response (Ball, Stein, Ramsawh, Campbell‐Sills, & Paulus, 2014; Lueken et al., 2016). Most studies used only one or two of these modalities to predict treatment response, and none (to the knowledge of the authors) so far combined functional and structural MRI (fMRI, sMRI, respectively) in addition to CD data to predict BPD therapy response.

Such a multimodal and integrative approach to predict treatment outcome is of major interest in a disorder with a psychotherapy response rate not significantly exceeding 50% of patients (Stoffers et al., 2012). It is also in line with the leading idea of the Research Domain Criteria (RDoC) Initiative, which is to ground treatment development and outcome prediction on dimensions of equally weighted neurobiological measures and behavioral functions (Cuthbert & Kozak, 2013).

Affect dysregulation is a central characteristic of BPD psychopathology (Linehan, 1993) and a main target of intervention across various psychotherapeutic programs. Meanwhile, neuronal correlates of affect dysregulation in BPD have been identified with a meta‐analysis suggesting multimodal (functional and structural) dysfunctions in frontolimbic brain areas, such as left amygdala, right parahippocampus, left hippocampus as well as left and right hemisphere inferior and superior frontal, temporal, parietal, and motor‐associated regions as well as cerebellar vermis (Schulze, Schmahl, & Niedtfeld, 2016). Therefore, we specifically examined the role of functional and structural alterations of these regions of interest (ROIs) in predicting treatment response of patients with BPD receiving a psychosocial treatment—dialectic behavior therapy (DBT)—which specifically focuses on improving affect regulation capacity in patients with BPD (Kliem et al., 2010; Stoffers et al., 2012).

Among the various techniques proposed for predicting treatment response via neurobiological markers (Lueken et al., 2016), the random forest method (Breiman, 2001) stands out for its robustness and excellent suitability for predictive data analysis (Qi, Bar‐Joseph, & Klein‐Seetharaman, 2006). The overall procedure can be summarized in three main steps: decision tree and forest building, cross‐validation, and selection of most relevant variables to build a final random forest model (further details can be found in (Ball et al., 2014; Breiman, 2001; Bureau et al., 2005; Genuer, Poggi, & Tuleau‐Malot, 2010; Strobl, Malley, & Tutz, 2009)). With regard to psychotherapeutic outcome predictions, random forest models have already successfully been used with fMRI (Ball et al., 2014) and sMRI data separately (Wade et al., 2015), but (to the knowledge of the authors) not with multimodal fMRI and structural MRI (sMRI) data sets in addition to CD data.

Here, we used random forests based on CD, fMRI, and sMRI data to specifically predict DBT outcomes in patients with BPD. FMRI data were based on three different affect regulation tasks which had been acquired in addition to sMRI data from 31 patients with BPD before DBT (Niedtfeld et al., 2017; Schmitt, Winter, Niedtfeld, Herpertz, & Schmahl, 2016; Winter et al., 2017). In summary, these longitudinal studies showed that successful DBT is behaviorally represented in a more efficient emotion regulation during reappraisal of negative pictures, a normalization of the processing of painful stimuli, and lower emotional susceptibility during distraction via alterations of the respective functional networks (including bilateral parahippocampus, amygdala, anterior cingulate cortex, orbitofrontal cortex as well as right dorsolateral prefrontal cortex, and cerebellum).

We expected that the multimodally affected regions (see erratum on Schulze et al., 2016, table 3 “multimodally affected brain regions in patients with BPD” (Schulze et al., 2016)) would provide useful information for building accurate predictive models. Therefore, we examined the utility of random forest analysis to specifically predict treatment response to DBT in patients with BPD via multimodal data sets in addition to CD data.

## 2 MATERIALS AND METHODS

### 2.1 Participants

The sample used in this study comprised 31 female patients drawn from previous studies (Niedtfeld et al., 2017; Schmitt et al., 2016; Winter et al., 2017) meeting DSM‐IV criteria for BPD diagnosis (including affective instability and self‐injurious behavior) who received treatment in two residential DBT programs at the Center for Psychosocial Medicine and the Central Institute of Mental Health, both located at Heidelberg University and providing fMRI data from all three tasks and sMRI data.

As in the three earlier reports using the patient pool (Niedtfeld et al., 2017; Schmitt et al., 2016; Winter et al., 2017), exclusion criteria were left‐handedness, traumatic brain injury, lifetime diagnoses of schizophrenia or bipolar I disorder, mental or developmental disorders, substance dependence during the last year, consumption of illegal drugs in the last two months, current severe depressive episode, and benzodiazepine use. Furthermore, patients who had significant DBT skills training experience and/or did not meet criteria for MRI safety and eligibility were excluded.

Since the same patient pool as in the three earlier reports (Niedtfeld et al., 2017; Schmitt et al., 2016; Winter et al., 2017) was used, also clinical and demographical measures were adopted: Trained clinical psychologists assessed BPD diagnoses using the International Personality Disorder Examination (IPDE; Loranger, 1999) and Axis I disorders using the Structured Clinical Interview for DSM‐IV (SCID‐I; Wittchen, Wunderlich, Gruschwitz, & Zaudig, 1997). Accompanying the MRI measurements, BPD symptom severity was assessed using the Zanarini Rating Scale for BPD (ZAN‐BPD; Zanarini et al., 2003) and the Borderline Symptom List (BSL; Bohus et al., 2007). Emotion regulation difficulties were assessed by the Difficulties in Emotion Regulation Scale (DERS; Gratz & Roemer, 2004) and dissociative symptoms by a self‐report questionnaire (FDS; German version of the Dissociative Experiences Scale DES; Spitzer et al., 1998). The State‐Trait Anxiety Inventory (STAI‐state, STAI‐trait; Spielberger, Gorusch, & Lushene, 1970) was used to probe anxiety of the patients, depressiveness was measured via the Beck Depression Inventory (BDI; Beck, Ward, Mendelson, Mock, & Erbaugh, 1961), and participants were instructed to memorize digits to estimate working‐memory capabilities (digit span; Tewes, 1991). Additionally, patients were asked, if they had used skills within the last three days and if they considered this skill use as successful. Identification of DBT responders was performed as in the three earlier reports (reliable change index [Jacobson & Truax, 1991] based on the symptom reduction in the ZAN‐BPD total score, cut‐off ≥ 1.96; Niedtfeld et al., 2017; Schmitt et al., 2016; Winter et al., 2017) resulting in an identification of 16 patients showing a significant improvement after therapy. Table 1 summarizes clinical and demographic measures and statistics of responder–nonresponder comparisons.

**Table 1:** Clinical and demographic measures

	All (*n* = 31; *SD*)	Responders (*n* = 16; *SD*)	Nonresponders (*n* = 15; *SD*)	Statistic (*df*)	All (*n* = 31; *SD*)	Responders (*n* = 16; *SD*)	Nonresponders (*n* = 15; *SD*)	Statistic (*df*)
*Pretreatment*					*Post‐treatment*			
Age in years	27.8 (7.5)	27.5 (7.4)	28.1 (7.8)	*t*(29) = 0.23				
Education in years^a^	11.1 (1.7)	10.6 (1.5)	11.5 (1.9)	*t*(29) = 1.51				
Medication in percent	48.4% (50.8%)	50% (51.6%)	46.7% (51.6%)	*t*(29) = 0.18				
Digit‐span	13.9 (3.2)	14.2 (2.6)	13.6 (3.8)	*t*(29) = 0.45	15.2 (3.4)	14.6 (2.3)	15.7 (4.3)	*t*(29) = 0.96
BDI^a^	29.0 (8.0)	31.5 (6.0)	26.3 (9.2)	*t*(29) = 1.86	22.2 (11.4)	20.9 (11.6)	23.7 (11.4)	*t*(29) = 0.67
BSL^a^	2.1 (0.6)	2.3 (0.4)	1.8 (0.6)	*t*(29) = 2.65*	1.7 (0.8)	1.7 (0.9)	1.7 (0.8)	*t*(29) = 0.11
DERS	132.1 (22.6)	134.9 (19.0)	129.2 (26.3)	*t*(29) = 0.70	108.0 (23.8)	108.0 (22.1)	108.1 (26.2)	*t*(29) = 0.03
FDS	22.4 (13.5)	26.5 (13.2)	18.1 (12.7)	*t*(29) = 1.79	22.2 (14.1)	25.0 (15.5)	19.3 (12.2)	*t*(29) = 1.13
STAI‐state	57.8 (9.5)	60.1 (6.0)	55.5 (11.9)	*t*(29) = 1.38	54.5 (11.6)	53.3 (11.9)	55.7 (11.5)	*t*(29) = 0.58
STAI‐trait	62.8 (7.3)	65.4 (6.2)	60.0 (7.4)	*t*(29) = 2.21*	57.1 (9.1)	57.2 (10.5)	57.1 (7.6)	*t*(29) = 0.03
ZAN^a^	16.4 (5.8)	19.4 (4.3)	13.1 (5.6)	*t*(29) = 3.59**	10.1 (6.4)	8.3 (5.6)	11.9 (6.9)	*t*(29) = 1.61

Note

Values presented as mean and standard deviation (*SD*). Statistic refers to comparison between responders and nonresponders as *t*‐value and degrees of freedom (*df*). Medication at pretreatment was missing six data points (three from responders), which were predicted via decision tree. The scores of the Beck Depression Inventory (BDI) at post‐treatment were missing one data point (responder), which was predicted via decision tree.

Abbreviations: BSL, Borderline Symptom List; DERS, Difficulties in Emotion Regulation Scale; FDS, German version of the Dissociative Experiences Scale; STAI‐state, STAI‐trait, State‐Trait Anxiety Inventory; ZAN, Zanarini Rating Scale for borderline personality disorder.

a Variable was selected in any of the final models including clinical and demographic measures.

* *p* < .05.

** *p* < .0025 (Bonferroni corrected for 20 comparisons).

This study was approved by the local ethics boards of Mannheim and Heidelberg, Germany, and conducted according to the Declaration of Helsinki. After complete explanation of the study, written informed consent was provided. The study was part of a larger project on the neural correlates of DBT in BPD, registered as a clinical trial (German Clinical Trial registration, registration ID DRKS00000778).

### 2.2 Dialectical behavior therapy

Patients participated in a well‐established and evaluated 12‐week standard residential DBT‐based treatment (Bohus et al., 2004). The program comprised weekly skills training groups (emotion regulation skills, mindfulness, self‐esteem, and social competence) and individual treatment twice a week. Therapists were experienced Ph.D., M.D., and M.Sc. level clinicians (psychologists and physicians) and were supervised regularly. The treatment program has already been described in Schmitt et al. (2016), Niedtfeld et al. (2017) and Winter et al. (2017).

### 2.3 Cognitive reappraisal task

The cognitive reappraisal task (reap) is described in detail in Schmitt et al. (2016). In brief, participants were instructed for 2 s to either look at or decrease their emotions via cognitive reappraisal during the presentation of the following image. The following negative or neutral images were selected from the International Affective Picture System (Lang, Bradley, & Cuthbert, 1997) or the Emotional Picture Set (Wessa et al., 2010) and presented for 6 s. Negative images were low in valence and high in arousal. Neutral images had intermediate valence and low arousal. After the picture presentation, the letter “O” was presented occasionally (for 2 s) and participants had to respond to that by pressing a button as fast as possible. After each trial, a fixation cross was presented for 3–8 s (mean 5.5 s). In total, the experiment comprised 72 trials (18 per condition).

### 2.4 Sensory distraction task

The sensory distraction task (pain) is described in detail in Niedtfeld et al. (2017). In brief, participants were instructed to look at the following image for 2 s. The following negative or neutral images were selected from the International Affective Picture System or the Emotional Picture Set and presented for 6 s. Similar to the cognitive reappraisal task, negative images were low in valence and high in arousal and neutral images had intermediate valence and low arousal. During 50% of the presented images, a painful heat stimulus was delivered to the participants (individually adapted temperature). After the picture presentation, the letter “O” was presented occasionally (for 2 s) and participants had to respond to that by pressing a button as fast as possible. After each trial, a fixation cross was presented for 3–8 s (mean 5.5 s). In total, the experiment comprised 72 trials (18 per condition).

### 2.5 Cognitive distraction task

The cognitive distraction task (distr) is described in detail in Winter et al. (2017). In brief, participants were instructed to either look at or memorize five consonants, presented for 2 s, followed by a negative or neutral image (presented for 6 s) selected from the International Affective Picture System or the Emotional Picture Set. As in the other tasks, negative images were low in valence and high in arousal and neutral images had intermediate valence and low arousal. In the memorize condition, participants had to press a respective button as fast as possible to indicate whether a presented character (presented for 2 s) was included in the initially presented string after presentation of the image. In the view condition, the letter “O” was presented after the image (presented for 2 s) and participants had to respond to that by pressing a button as fast as possible. After each single‐letter presentation, a fixation cross was presented for 3–8 s (mean 5.5 s). In total, the experiment comprised 72 trials (18 per condition).

### 2.6 MRI data acquisition

Whole‐brain fMRI data was acquired on a 3 Tesla Siemens TRIO‐MRI (Siemens) with a 32‐channel head coil. The EPI sequence (same for all three functional tasks) comprised 36 slices (FOV = 192 mm, voxel size 3 × 3 × 3 mm, voxel matrix 64 × 64, flip angle = 80°, TE = 30 ms, TR = 2,000 ms).

Additionally, high‐resolution anatomical data (sMRI) were acquired (MPRAGE, T1‐weighted contrast, FOV = 256 mm, voxel size 1 × 1 × 1 mm, flip angle = 15°, TE = 2.75 ms, TR = 1,570 ms).

### 2.7 Image processing

FMRI data were processed using SPM8 (http://www.fil.ion.ucl.ac.uk/spm/) as described in the corresponding reports, reap: Schmitt et al. (2016); pain: Niedtfeld et al. (2017); distr: Winter et al. (2017). Preprocessing comprised slice timing correction, spatial realignment, coregistration onto the participants' segmented high‐resolution scan, normalization into MNI space, and spatial smoothing. On single‐subject level, a general linear model per time point using separate block regressors for the presentation time of each picture in the four experimental conditions (6 s each) was applied. To correct for global signal intensity variation and low‐frequency fluctuations, a high‐pass filter of 128 s cut‐off was applied.

SMRI data were processed using CAT12 (http://www.neuro.uni-jena.de/cat/) implemented in SPM12 (http://www.fil.ion.ucl.ac.uk/spm/). Preprocessing comprised data segmentation (adjusted for writing gray matter volumes [GMV] according to the Automated Anatomical Labeling Atlas [AAL; Tzourio‐Mazoyer et al., 2002]); note that, to parallelize data with Schulze et al. (2016), the GMV for the vermic lobule IV/V of both hemispheres was summarized to a bilateral volume. Data quality was checked visually and by sample homogeneity using CAT12.

ROIs were selected according to Schulze et al. (2016), using multimodally (smaller or higher GMV and enhanced or decreased activation) affected brain regions in patients with BPD in comparison with healthy controls (see erratum on Schulze et al., 2016, table 3 “Multimodally Affected Brain Regions in Patients With BPD”). Specifically, these ROIs were as follows: left amygdala, right inferior frontal gyrus pars opercularis, right inferior frontal gyrus pars triangularis, right superior frontal gyrus, left hippocampus, right parahippocampus, left precentral gyrus, left precuneus, right supplementary motor area, left middle temporal gyrus, right superior temporal gyrus, and bilateral cerebellar vermis IV/V. For extraction of Fmri activations, respective masks were created using the AAL implemented in the WFU Pickatlas (Maldjian, Laurienti, Kraft, & Burdette, 2003) and mean single‐subject activation was extracted for the contrasts of watching negative versus neutral pictures (emotional challenge) and regulating negative versus watching negative pictures (regulation) in the three tasks using MarsBaR (Brett, Anton, Valabregue, & Poline, 2002). GMV for these ROIs were derived from the.xml files written by CAT12.

### 2.8 Statistical analysis

To predict therapy response to DBT using CD, fMRI, and sMRI data, we performed a random forest classification approach, following the methodological (or statistical) procedure presented in Ball et al. (2014). To do so, the *randomForest* package was used in R statistics (version 3.5.1, http://cran.r-project.org). Our main objective was to predict responder status using CD, functional activations, and GMV extracted from anatomical ROIs (functional and anatomical ROI's were selected from the meta‐analysis by Schulze et al. (2016), as described under “Image processing”) during the three experiments (reap, pain, and distr) in two differential contrasts (emotional challenge and regulation), and the anatomical images at the start of treatment.

**Figure 1:**
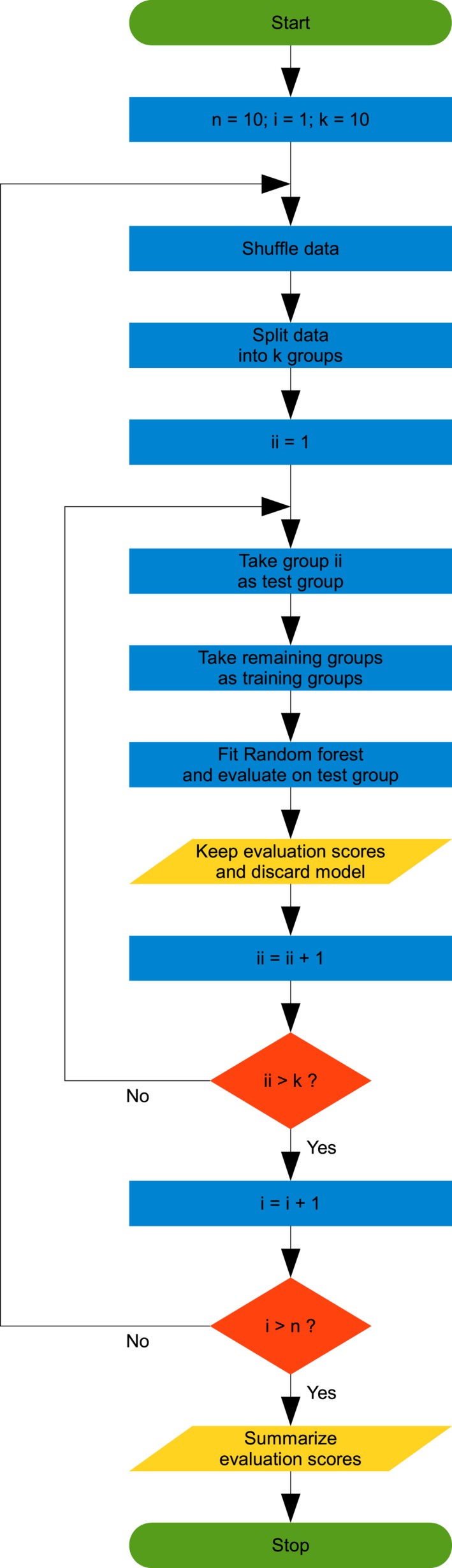
Flowchart of the 10‐repeated 10‐fold cross‐validation procedure. Green round‐edged boxes show start and stop of the procedure, blue boxes show operations, yellow parallelograms show output, and orange diamonds show branches

In contrast to Ball et al. (2014), we used 10,000 classification trees in the random forest procedure and performed a 10‐repeated 10‐fold cross‐validation as described at https://machinelearningmastery.com/k-fold-cross-validation/. The procedure was carried out as follows: First, the order of the subjects was shuffled. In the second step, the data were split into *k* groups (here: *k* = 10, while groups one to nine comprised three subjects and group 10 comprised four subjects). In step three, the following algorithm was performed for each unique group: (1) Take the group as test group; (2) the remaining groups build up the training group; (3) fit the random forest model (each tree using a bootstrapped subsample of the model training group and a randomly selected subset of the seven sets of independent variables (CD, fMRI, sMRI, and all combinations of these sets)) and evaluate it on the test group; 4. keep the evaluation scores and discard the model. In the fourth step, the evaluation scores were summarized. The whole procedure was repeated 10 times for each set of the independent variables, resulting in a different split of the sample during each repetition, and evaluation scores were summarized over the 10 repetitions (see Figure 1). The calculated median permutation importance scores (Genuer et al., 2010) were then used to select variables for inclusion in the final models. Variables survived the selection process, if the median permutation importance was greater than the absolute value of the most negative value and *t* test showed significant difference to zero (Ball et al., 2014). After the variables for the final models were identified, the described 10‐repeated 10‐fold cross‐validation procedure was applied onto the final models. Afterward, the corresponding mean values were used to evaluate classification accuracy, sensitivity, specificity, positive, and negative likelihood ratios (each subject was classified as responder or nonresponder 70 times (=seven models × 10 repetitions)).

The response rate for DBT was 51.61% (95% CI: 32.98%–70.25%), and therefore, a simple all‐respond model, that is, classifying all patients as responders would achieve 51.61% accuracy. Consequently, only models performing better than 70.25% perform statistically better than the all‐respond model and therefore provide a significant gain in prediction of DBT treatment outcome.

To aid in the interpretation of the selected ROIs, correlations were performed between the ROIs (fMRI and sMRI) and the CD measurements in the final models.

## 3 RESULTS

Table 1 summarizes CD measures and statistics of responder—nonresponder comparisons. Effects of fMRI tasks, behavioral data, and relationships between brain activation and symptom severity have already been reported (Niedtfeld et al., 2017; Schmitt et al., 2016; Winter et al., 2017). Here, we report the initial (all variables) and the final model (subset of variables contributing most to classification accuracy) for each set of predictor variables. All permutation importance scores chosen for the final models significantly differed from zero (*p* < .001).

### 3.1 CD model

The original CD model comprised the following predictors: age in years, education in years, medication at start of therapy (yes/no), digit span, BDI total score, BSL total score, FDS total score, DERS total score, STAI‐state total score, STAI‐trait total score, and ZAN‐BPD total score. Four predictors entered the final model (ordered by predictive value): ZAN‐BPD total score, BSL total score, education in years, and BDI total score. Accuracy of the final model was 68.00% (Table 2) and therefore not significantly better than a simple all‐respond model.

**Table 2:** Test characteristics of all final models

Model	Mean accuracy (%)	Mean sensitivity	Mean specificity	Mean LR+ (ub, lb 95% CI)	Mean LR− (ub, lb 95% CI)
CD	68.00	0.77	0.61	2.00 (2.34, 1.65)	0.37 (1.37, −0.63)
CD and sMRI	73.33	0.74	0.74	2.88 (3.21, 2.54)	0.35 (1.49, −0.80)
CD and fMRI and sMRI	73.50	0.73	0.75	2.91 (3.26, 2.57)	0.36 (1.46, −0.75)
CD and fMRI	74.75	0.75	0.72	2.74 (3.10, 2.39)	0.35 (1.39, −0.70)
fMRI	75.92	0.76	0.76	3.14 (3.48, 2.80)	0.31 (1.45, −0.82)
sMRI	75.92	0.72	0.81	3.71 (4.06, 3.37)	0.34 (1.52, −0.83)
fMRI and sMRI	76.08	0.77	0.78	3.54 (3.85, 3.24)	0.29 (1.59, −1.00)

Abbreviations: CD, clinical and demographic; fMRI, functional magnetic resonance imaging; LR−, negative likelihood ratio; LR+, positive likelihood ratio; sMRI, structural magnetic resonance imaging; ub, lb 95% CI, upper and lower bound of the 95% confidence interval.

### 3.2 Combined CD and sMRI model

The original combined CD and sMRI model comprised all predictors used in the original CD and the original sMRI model (see below). Five predictors entered the final model (ordered by predictive value): ZAN‐BPD total score, BSL total score, left amygdala (GMV), and BDI total score. Accuracy of the final model was 73.33% (Table 2) and therefore significantly better than a simple all‐respond model.

### 3.3 Combined CD, fMRI, and sMRI model

The original combined CD, fMRI and sMRI model comprised all predictors used in the original CD, fMRI (see below), and the initial sMRI model (see below). Six predictors entered the final model (ordered by predictive value): ZAN‐BPD total score, left amygdala (reap, regulation), left amygdala (reap, emotional challenge), right parahippocampus (reap, regulation), BSL total score, and left amygdala (GMV). Accuracy of the final model was 73.50% (Table 2) and therefore significantly better than a simple all‐respond model.

### 3.4 Combined CD and fMRI model

The original combined CD and fMRI model comprised all predictors used in the original CD and the original fMRI model (see below). Five predictors entered the final model (ordered by predictive value): ZAN‐BPD total score, left amygdala (reap, regulation), left amygdala (reap, emotional challenge), right parahippocampus (reap, regulation), and BSL total score. Accuracy of the final model was 74.75% (Table 2) and therefore significantly better than a simple all‐respond model.

### 3.5 FMRI model

The original fMRI model comprised mean activation for the emotional challenge and regulation contrasts in the three tasks (reap, pain, and distr) before treatment in the multimodally affected ROIs selected according to Schulze et al. (2016). Three predictors entered the final model (ordered by predictive value): left amygdala (reap, regulation), left amygdala (reap, emotional challenge), and right parahippocampus (reap, regulation). Accuracy of the final model was 75.92% (Table 2) and therefore significantly better than a simple all‐respond model.

### 3.6 SMRI model

The original sMRI model comprised GMV within the multimodally affected ROIs selected according to Schulze et al. (2016). Only left amygdala (GMV) entered the final model. Accuracy of the final model was 75.92% (Table 2) and therefore significantly better than a simple all‐respond model.

### 3.7 Combined fMRI and sMRI model

The original combined fMRI and sMRI model comprised all predictors used in the original fMRI and the original sMRI model. Three functional (but no structural) predictors entered the final model (ordered by predictive value): left amygdala (reap, emotional challenge), left amygdala (reap, regulation), and right parahippocampus gyrus (reap, regulation). Accuracy of the final model was 76.08% (Table 2) and therefore significantly better than a simple all‐respond model. Noteworthy, the final model comprised the same predictors as the fMRI model, but in a different order. Therefore, the slight improve of the mean accuracy might be a statistical variation due to the bootstrapped subsampling and random feature selection for each tree in the random forest procedure.

### 3.8 Model comparisons

**Figure 2:**
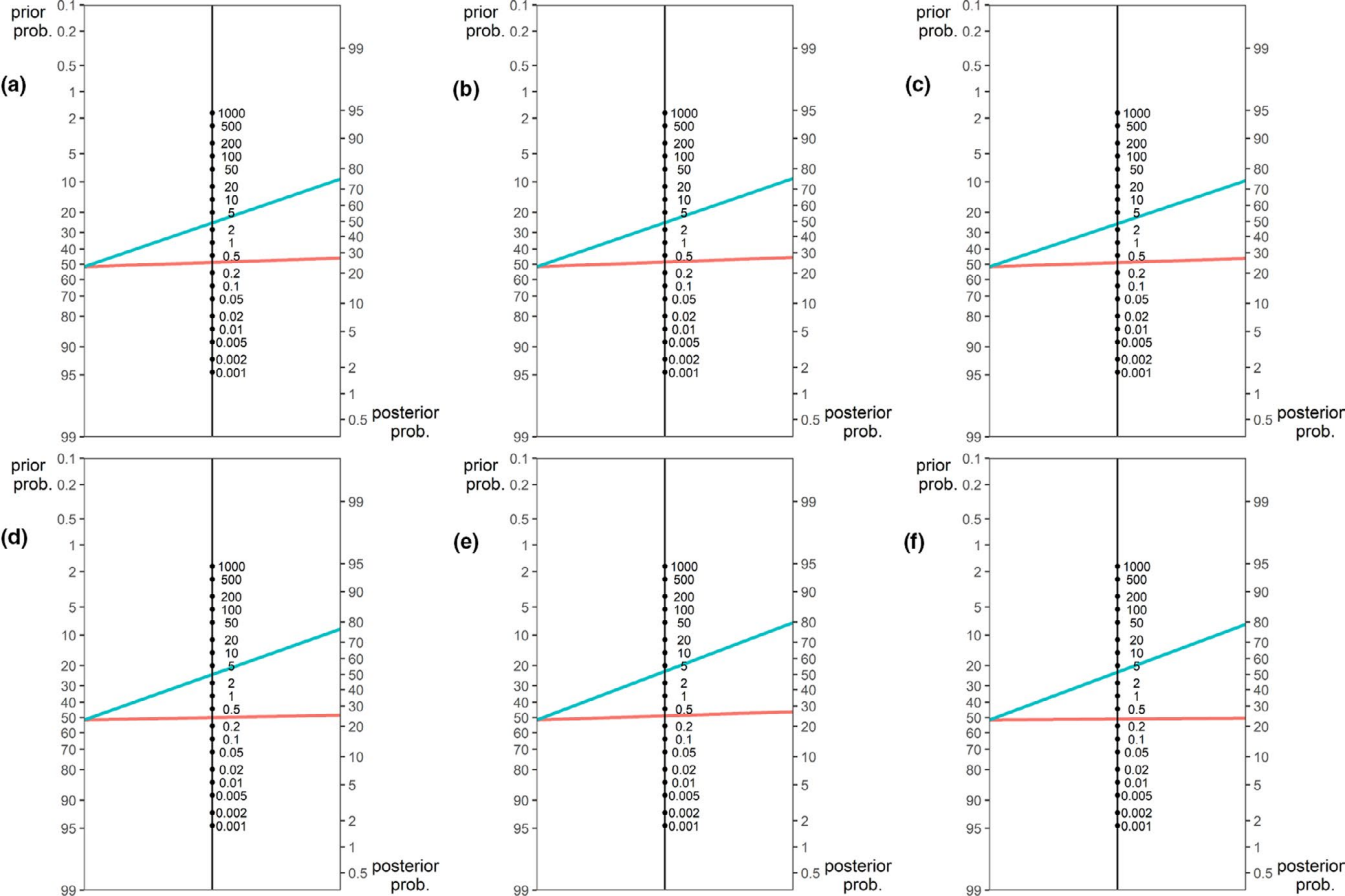
Comparison of positive and negative likelihood ratios (middle vertical line each) for the three models performing better than response rate. (a) CD and sMRI, (b) CD and fMRI and sMRI, (c) CD and fMRI (d) fMRI, (e) sMRI, and (f) fMRI and sMRI. Blue lines indicate positive, red lines negative test result (i.e., predicted responders and nonresponders, respectively)

**Figure 3:**
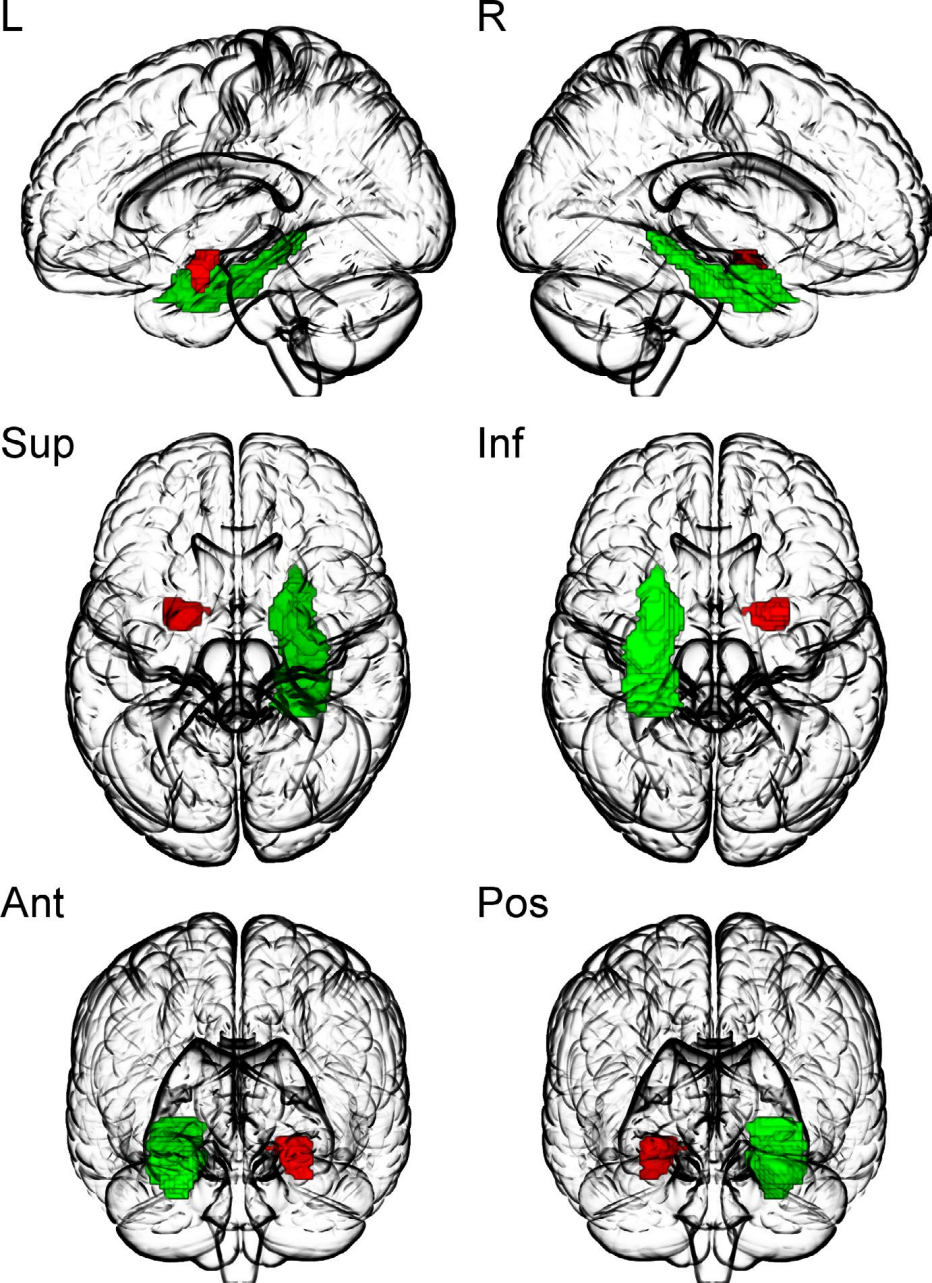
Localization of the brain areas included in the final models. Left amygdala, (red, reap emotional challenge and regulation, GMV); right parahippocampus (green, reap regulation) provided sufficient predictive power to be included in any of the final models. L: left view, R: right view, Sup: superior view, Inf: inferior view, Ant: anterior view, Pos: posterior view. This figure was created using MRIcroGL (https://www.mccauslandcenter.sc.edu/mricrogl/home)

Table 2 shows the features of the seven final models, and Figure 2 shows the increase in predictive information of each model which performed significantly better than an all‐respond model. In terms of the mean accuracy, the fMRI model and the sMRI model did not differ, whereas the combined fMRI and sMRI model showed a good balance of the advantages of both models and resulted in best accuracy and lowest negative likelihood ratio. Left amygdala (reap, emotional challenge and regulation, GMV) and right parahippocampus (reap, regulation) showed sufficient predictive power to be included in any of the final models including fMRI or sMRI data. Localization and modalities with sufficient predictive power of these ROIs are summarized in Figure 3.

Twenty‐four of the 31 subjects were misclassified at least once in the 70 classifications of which five subjects (one responder) were misclassified in more than 51.61% of the tests. Two of these subjects were hard to classify (more than five misclassifications during the 10 repetitions of a model) if the model contained CD or fMRI data, one subject if the model contained CD or sMRI data, one if the model contained CD data, and one subject was hard to classify in all models. The hard to classify subjects did not differ from the remaining subjects in terms of clinical, demographical, or fMRI data (if corrected for multiple comparisons), but showed smaller GMV of the left middle temporal gyrus (*p* < .0005, surviving Bonferroni correction) than the remaining subjects.

Split up by the number of subjects which were misclassified at least once per model, 15 subjects were misclassified by the CD model, 13 by the combined CD and sMRI model, 12 by the sMRI model, the combined CD and sMRI model, and the combined CD, fMRI, and sMRI model, and nine by the fMRI and the combined fMRI and sMRI model.

### 3.9 Further investigation of the variables used in the final models

**Figure 4:**
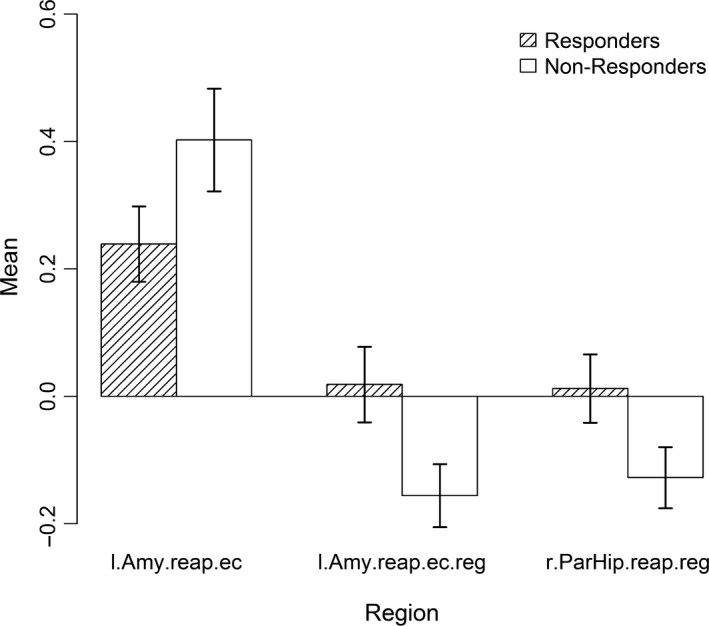
Mean fMRI activation in responders (shaded) and nonresponders (white) in regions selected for the final models comprising fMRI data for emotional challenge (negative watch vs. neutral watch) and regulation (negative regulate vs. negative watch). Error bars show standard error of the mean. l.Amy.reap.ec: left amygdala in the reappraisal task for emotional challenge, l.Amy.reap.reg: left amygdala in the reappraisal task for regulation, r.Par.Hip.reap.reg: right parahippocampus in the reappraisal task for regulation

**Figure 5:**
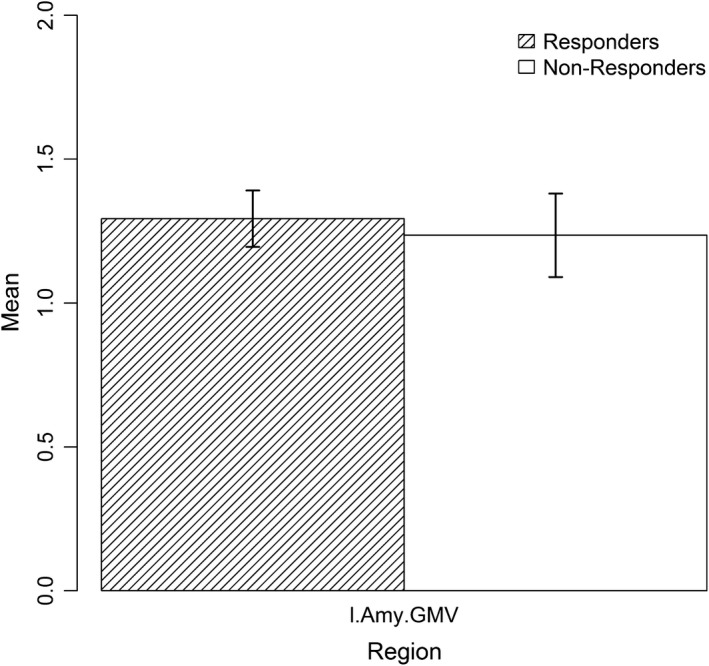
Mean GMV in responders (shaded) and nonresponders (white) in regions selected for the final models. Error bars show standard error of the mean. l.Amy.GMV: gray matter volume of the left amygdala

Figure 4 shows that responders had lower mean activation in the ROIs used in any final model during emotional challenge and greater mean activation during regulation. Regarding GMV, no mentionable difference was found between the groups (see Figure 5).

**Figure 6:**
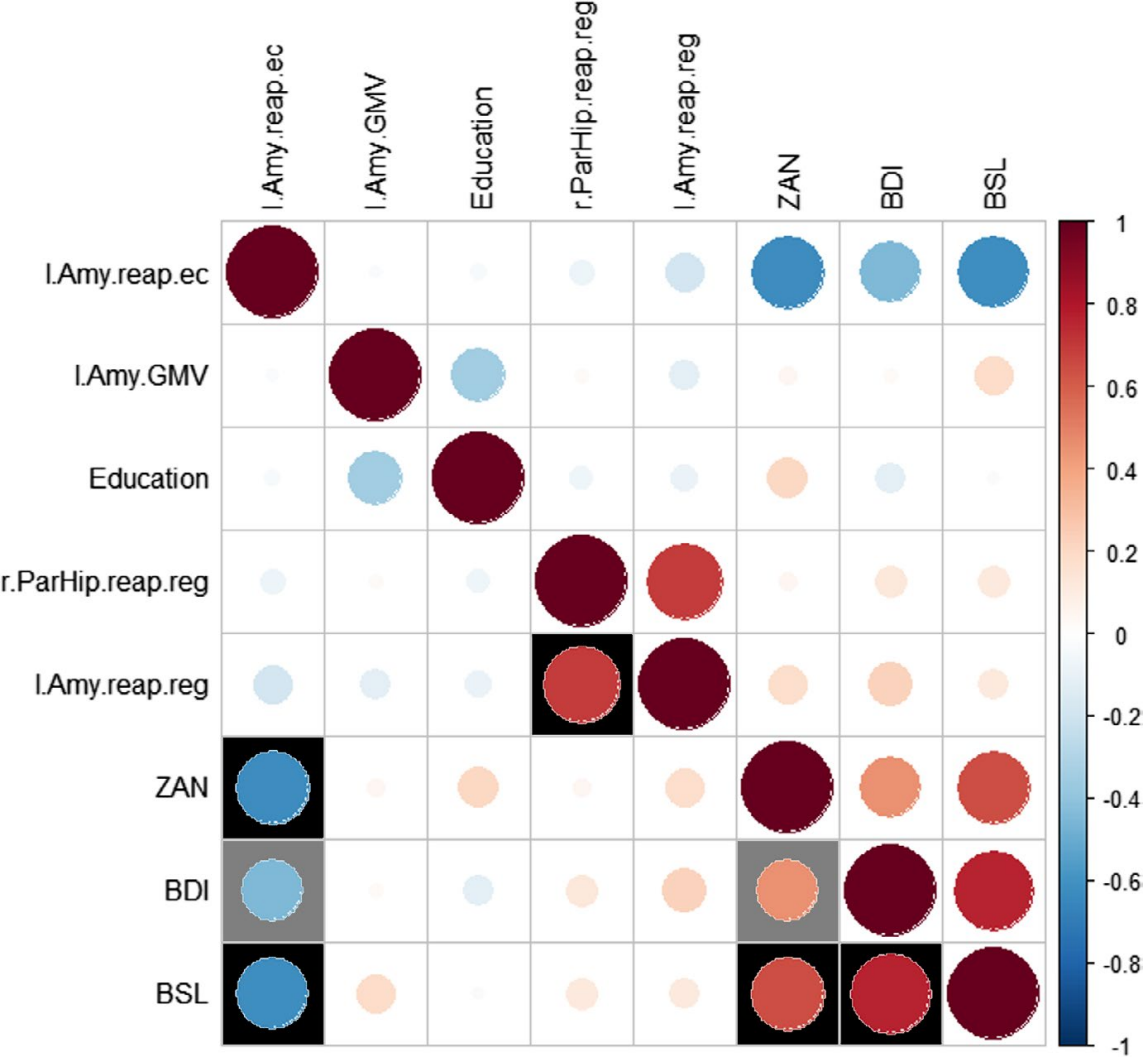
Correlations (Spearman) between all variables used in the final models. Positive correlations are displayed in red, and negative correlations are displayed in blue. Black fillings indicate significant (*p* < .0018, Bonferroni corrected) results, gray fillings indicate statistical trends (*p* < .05, not surviving Bonferroni correction). BDI, BDI total score; BSL, BSL total score; Education, education in years; l.Amy.GMV, gray matter volume of the left amygdala; l.Amy.reap.ec, left amygdala in the reappraisal task for emotional challenge; l.Amy.reap.reg, left amygdala in the reappraisal task for regulation; r.ParHip.reap.reg, right parahippocampus in the reappraisal task for regulation; ZAN, ZAN‐BPD total score

Correlations (Spearman's *ρ*; Bonferroni corrected: *p* < .0018 and uncorrected for multiple comparisons: *p* < .05) between all variables used in the final models (Figure 6) showed clustered positive correlations between clinical measures and negative correlations between left amygdala function during cognitive reappraisal for emotional control and clinical measures. Also, a positive correlation between left amygdala function and right parahippocampus function during cognitive reappraisal for regulation was found.

## 4 DISCUSSION

In this study, we used random forest models based on clinical/demographical data, functional, and structural neuroimaging data to predict DBT treatment response in patients with BPD. To our knowledge, this is the first study combining CD, fMRI, and sMRI data in random forest models for treatment outcome predictions. Our results support the conclusion of Ball et al. (2014), that fMRI data can be used to generate predictions with reasonable test characteristics. Furthermore, we show proof of principle that the combination of data from multiple modalities yields the potential to improve predictions.

The likelihood ratios indicate that relative to the odds of treatment response, predicted responders based on the combined fMRI and sMRI model (best model in terms of accuracy and negative likelihood ratio) are 3.54 times more likely to respond to DBT and predicted nonresponders are 3.45 times less likely to respond to DBT treatment. Based on the sMRI model (best model in terms of positive likelihood ratio), predicted responders are 3.71 times more likely to respond to DBT and predicted nonresponders are 2.94 times less likely to respond to DBT treatment.

All models except the CD model generated predictions that were significantly better than chance with accuracies of up to 76.08% (combined fMRI and sMRI model). The combined fMRI and sMRI model contained the same variables as the fMRI model (i.e., no sMRI data), but in a different order and should therefore be interpreted as a variation of the fMRI model. The models including CD data performed less well than the models without CD data (surprisingly the combined CD, fMRI, and sMRI model only reached rank five of seven). This shows on the one hand the importance of testing combinations of available modalities of data, since certain modalities might limit the full potential of others, if included in the same model. On the other hand, our results point toward the importance of MRI‐based data in mental health predictomics and personalized therapy (the CD‐only based model showed an accuracy of 68%). The finding that the CD‐only based model was not superior to the all‐respond model supports the RDoC approach that subgroups of psychiatric patients that derive from neurobiological data are more likely to predict therapy response than purely clinical data (Bzdok & Meyer‐Lindenberg, 2018; Cuthbert & Insel, 2013). According to our results, there is no clear winning model: The sMRI model wins by its simplicity (only GMV of the left amygdala as predictor) and feasibility in clinical routine (only one T1 MRI sequence to run and no time‐consuming task‐based data to collect and analyze), while the combined fMRI and sMRI model comprises the potential to utilize the advances of both modalities (some subjects were classified correctly in all tests by using fMRI‐based data but not sMRI‐based data and vice versa).

Brain activation during watching negative versus neutral images (emotional challenge) and regulating versus watching negative images (regulation) during the reappraisal task provided best predictive power, compared with the other tasks. Especially, the mean activation within left amygdala during the reappraisal task yielded superior predictive power, compared with the variables derived from the other tasks. Therefore and consistent with our expectation, especially left amygdala played a crucial role in predicting treatment response and showed functional differences between responders and nonresponders. From a prognostic point of view, hyperreactivity of the left amygdala during the emotional challenge condition in the reappraisal task points toward a lower chance to respond to DBT. This might imply that patients with lower emotional reactivity might be more likely to respond to DBT. Furthermore, higher cognitive functions such as regulating emotions might be trained during DBT. This is reflected in our finding that DBT responders show initially greater activation of the left amygdala during the regulation condition in the reappraisal task, reflecting lower regulation success. In addition, neuronal activity of the right parahippocampus, as a crucial part of the emotion regulation network (Frank et al., 2014), was found to be a significant predictor of treatment outcome and showed similar features of activation as the left amygdala.

Notably, there was no more significant difference in BSL and ZAN‐BPD total scores between responders and nonresponders post‐treatment. This might be explained by the fact that categorization into responders and nonresponders via the well‐established reliable change index (Jacobson & Truax, 1991) led to higher BPD symptom severity in the responder group (Schmitt et al., 2016). The pretreatment to post‐treatment differences, however, were greater in the responder group, which is likely to be based on a known phenomenon (Gratz, Dixon‐Gordon, & Tull, 2014; Schmitt et al., 2016).

Taken together, our findings (i.e., high predictive power of the mean activations in brain regions specifically connected with emotion regulation during the reappraisal task (Koenigsberg et al., 2009; Schulze et al., 2011) as well as GMV of the left amygdala) further highlight the relevance of emotion regulation skills in BPD psychopathology and its importance in psychotherapy of BPD. Correlations between the variables used in the final models showed positive correlations within clinical measures and between left amygdala and right parahippocampus function during regulation of the cognitive reappraisal task and negative correlations between clinical measures and the activation of left amygdala in the reappraisal task for emotional challenge. This indicates that clinical measures are intermingled with each other and suggests a link between brain activation and BPD symptoms (represented by the ZAN and BPD scores) being especially represented in the left amygdala during emotional challenge, but not during regulation. This finding points toward a less regulated communication between subcortical areas as a response to emotionally arousing stimuli (Hamann, 2001; Phelps, 2004) explicitly when patients are not trying to regulate their emotions. Moreover and contrary to all other models, the CD‐only based model did not perform significantly better than the all‐respond model, highlighting the importance of neurobiological measures in treatment response prediction.

## 5 LIMITATIONS

The main limitation of the present study is the small sample size. Therefore, the findings presented here should be interpreted as proof of principle and should not be read as suggestions for therapy selection until validated by studies using a larger sample size and models based on a wider scope of modalities. However, random forests use bootstrapping and should generate reliable predictions even in small samples and usually do not have the problems of overfitting (Ball et al., 2014; Breiman, 2001; Strobl et al., 2009). To further minimize the effects of small sample size and sample selection and increase stability of our results, we used a high number of classification trees and applied a 10‐repeated 10‐fold cross‐validation for variable selection and testing the final models. Still, the sample is too small to conduct model comparisons using traditional methods. Consequently, conclusions about model comparisons are limited and replication using a larger sample is highly desirable.

Also, there are further modalities and variables imaginable to enhance classification performance, such as additional questionnaires and ROI's, resting state fMRI, diffusion tensor imaging, PET, (electro‐) physiological, genomic, and baseline cortisol, or cortisol reactivity data.

Our study specifically tested treatment outcome prediction of DBT for BPD via random forest models. Nevertheless, there are various alternative psychotherapy options for BPD, which are not covered by our test for utility of random forest models in treatment outcome prediction for BPD per se, as presented here. Therefore, further studies should consider testing alternative treatments or combinations of treatments as well, to take a further step toward finding the best individualized treatment.

## 6 CONCLUSIONS

Here, we showed proof of principle that random forest models built with CD and multimodal MRI data can provide predictions of therapy response with reasonable test characteristics, outperforming models with CD data only. Our results suggest that fMRI and sMRI have a significant role in predicting treatment outcomes for DBT in patients with BPD. Future studies should examine if our findings remain valid after testing them in a larger sample, set an additional focus on the interplay between hard‐wired subcortical structures and higher cognitive cortical functions, and by continuing the use of the promising approach of random forest classifications or machine learning in general on alternative treatments, to develop predictive models with verified clinical relevance. Also, classifications might be further enhanced by including additional sets of variables as, for example resting state fMRI, diffusion tensor imaging, PET, (electro‐) physiological, genomic, and baseline cortisol, or cortisol reactivity data.

## CONFLICTS OF INTEREST

None declared.

## Data Availability

The data that support the findings of this study are available from the corresponding author upon reasonable request.
